# Sesquiterpenoids from the Rhizomes of *Homalomenaocculta*

**DOI:** 10.1007/s13659-016-0104-8

**Published:** 2016-07-05

**Authors:** Jun-Li Yang, Ya-Min Zhao, Yan-Ping Shi

**Affiliations:** Key Laboratory of Chemistry of Northwestern Plant Resources of CAS and Key Laboratory for Natural Medicine of Gansu Province, Lanzhou Institute of Chemical Physics, Chinese Academy of Sciences, Lanzhou, 730000 People’s Republic of China; State Key Laboratory of Applied Organic Chemistry, Lanzhou University, Lanzhou, 730000 People’s Republic of China

**Keywords:** *Homalomena occulta*, Sesquiterpenoids, Oplopananes

## Abstract

**Abstract:**

Naturally occurring oplopanane sesquiterpenoids are rarely reported. A phytochemical investigation on the rhizomes of *Homalomena occulta* (Lours) has resulted in the discovery of six oplopanane sesquiterpenoids (**1**–**6**), including four new (**1**–**4**) and one 3,5-*seco*-oplopanane (**6**), together with three previously reported sesquiterpenoids (**7**−**9**). In addition three new oplopananes (**2a**–**4a**) were also obtained by chemical transformation. All structures of these sesquiterpenoids were established based on the comprehensive spectroscopic analyses, including NMR, MS, and IR, and comparing with the literatures.

**Graphical Abstract:**

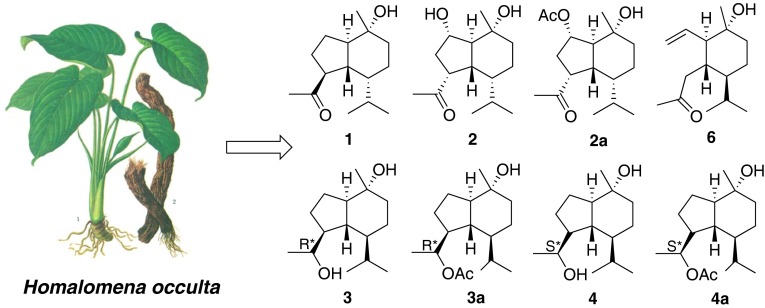

**Electronic supplementary material:**

The online version of this article (doi:10.1007/s13659-016-0104-8) contains supplementary material, which is available to authorized users.

## Introduction

With its rich cultural heritage and biodiversity, traditional Chinese medicine (TCM) has potential as a source for the discovery of structurally novel bioactive compounds. During the last twenty years, considerable efforts have been dedicated towards the exploration of the natural product chemistry of TCM. *Homalomena occulta* (Lour.) was officially listed in the Chinese Pharmacopoeia (named Qian-nian-jian) [1], and was found to occur in the tropical and sub-tropical areas of Asia and America. The plant has been used for the treatment of rheumatoid arthritis, trengthening tendons and bones, and invigorating the kidney and liver [2, 3]. Recent reports of *H. occulta* have also shown that the species is one of the most prolific sources of compounds with new structures [2–6].

Our research group has considerably focused on phytochemical investigations of TCM [7–10]. As part of our continuing efforts to obtain novel compounds with exquisite structural architectures, we initiated a chemical investigation of *H. occulta*, which has thus far led to the isolation and structural elucidation of six oplopanane sesquiterpenoids (**1**–**6**), including four new (**1**–**4**) and one 3,5-*seco*-oplopanane (**6**), together with three previously reported sesquiterpenoids (**7**−**9**).

## Results and Discussion

**Fig. 1:**
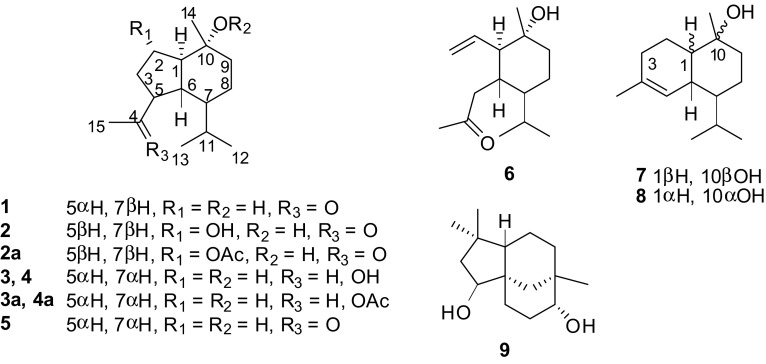
Molecular structures of sesquiterpenoids **1**−**9**

By means of diverse chromatographic methods, including silica gel and LH-20, four new oplopanane sesquiterpenoids (**1**−**4**) (Fig. 1) have been purified from an 88 % ethanol/water extract of the rhizomes of *H. occulta*.

Compound **1** was isolated as an optically active colorless oil with $[\alpha ]_{D}^{20}$ –54 (*c* 0.07, CHCl_3_). Its molecular formula was analyzed as same as oplopanone (**5**) [11], i.e. C_15_H_26_O_2_, based on a pseudomolecular ion peak at *m*/*z* 256.2272 [M + NH_4_]^+^ (calcd. 256.2271) in the positive HRESIMS spectrum, indicating three degrees of unsaturation. The IR absorptions suggested the existence of hydroxy (3382 cm^−1^) and ketone (1705 cm^−1^) functionalities. The ^13^C NMR spectra (Table 1) showed the presence of one oxygenated quaternary carbon (*δ*_C_ 73.2) and one ketone carbon (*δ*_C_ 212.3). Overall NMR pattern, together with the established molecular formula, showed close resemblance to those of oplopanone (**5**) [11], which indicated that compounds **1** and **5** were possibly epimers of each other. The large coupling constants of *J*_H-1,H-6_ (12.0 Hz) and *J*_H-5,H-6_ (10.4 Hz) indicated the *trans* relationship from H-1 to H-6, and from H-5 to H-6, while the small coupling constants of *J*_H-6,H-7_ (4.0 Hz) suggested H-7 as *β*-oriented. A molecular modeling study based on no NOE correlation from H-1 to H_3_-14 was used to determine the *β*-oriented Me-14. Finally, compound **1** was 6-*epi*-oplopanone.

**Table 1:** NMR spectroscopic data for compound **1** in CDCl_3_

Position	*δ*_H_ (*J* in Hz)	*δ*_C_	Position	*δ*_H_ (*J* in Hz)	*δ*_C_
**1**	2.07, ddd (6.8, 12.0, 12.0)	52.5 (d)	**8a** **8b**	1.55, m 1.01, dd (3.2, 13.6)	23.0 (t)
**2a**	1.90, m	24.7 (t)	**9a**	1.77, dt (3.2, 12.4)	42.2 (t)
**2b**	1.42, m		**9b**	1.40, m	
**3a**	1.94, dt (2.8, 6.0)	27.5 (t)	**10**		73.2 (s)
**3b**	1.58, m				
**4**		212.3 (s)	**11**	1.67, m	29.2 (d)
**5**	3.12, ddd (3.2, 10.4, 10.4)	50.5 (d)	**12 and 13**	0.78, d (7.2) 0.88, d (7.2)	15.8 (q) 21.3 (q)
**6**	1.35, ddd (4.0, 10.4, 12.0)	49.1 (d)	**14**	1.09, s	19.4 (q)
**7**	1.59, m	42.7 (d)	**15**	2.15, s	31.5 (q)

Compound **2**, obtained as an optically active colorless oil with $[\alpha ]_{D}^{20}$ +20 (*c* 0.1, CHCl_3_), was assigned a molecular formula C_15_H_26_O_3_ as analyzed from a pseudomolecular ion at *m*/*z* 277.1779 [M + Na]^+^ (calcd. 277.1774) in its positive HRESIMS, indicative of three degrees of unsaturation. Its IR spectrum showed absorptions for hydroxy (3392 cm^−1^) and ketone (1704 cm^−1^) groups. A spectroscopic analogy of **2** with **1** indicated that compound **2** was an oplopanane-type sesquiterpenoid. The significant variations of **1** and **2** in their ^1^H NMR spectra were the disappearance of methylene signals of H_2_-2 (*δ*_H_ 1.90/1.42) in **1** and the appearance of a multiplet at *δ*_H_ 4.10 in **2**. These changes could be explained by the presence of a hydroxy group at C-2 in compound **2**, which was further elucidated from an HMBC experiment. In confirmation of the relative configuration, **2** gave a 2-monoacetate (**2a**) (Table 2) after overnight treatment with Ac_2_O/Py (Sect. 3). As in **2a**, the coupling constants *J*_1,6_ = 12.4 Hz, *J*_1,2_ = 9.2 Hz, *J*_5,6_ = 2.4 Hz, and *J*_6,7_ = 8.9 Hz allowed the placement of H-5, H-6, and H-7 on *α*-orientation, and H-1 and OH-2 on *α*-orientation. The NOE correlations of H_3_-14 with H-2/H-6, and H-5 with H-2/H-7 were used to determine H_3_-14 as *β*-oriented. Thus, the structure of **2** was established as 5,7-*diepi*-2*α*-hydroxyoplopanone.

**Table 2:** NMR spectroscopic data for compounds **2**, **2a**, and **3** in CDCl_3_

Position	**2**		**2a**		**3**	
	*δ*_H_ (*J* in Hz)	*δ*_C_	*δ*_H_ (*J* in Hz)	*δ*_C_	*δ*_H_ (*J* in Hz)	*δ*_C_
**1**	1.50, m	60.4 (d)	1.75, dd (9.2, 12.4)	59.9 (d)	1.42, dt (4.8, 11.6)	57.5 (d)
**2a**	4.10, m	72.2 (d)	5.08, ddd (5.6, 8.8, 9.2)	74.0 (d)	1.78, dt (4.8, 12.4)	25.4 (t)
**2b**					1.18, m	
**3a**	1.82, m	36.9 (t)	2.08, m	35.8 (t)	1.75, m	22.5 (t)
**3b**	1.82, m		1.93, m		1.58, m	
**4**		210.9 (s)		210.0 (s)	3.98, q (6.4)	68.6 (d)
**5**	1.75, m	42.2 (d)	2.79, ddd (2.4, 8.0, 10.0)	44.1 (d)	1.72, m	48.9 (d)
**6**	2.73, m	52.6 (d)	1.86, ddd (2.4, 8.9, 12.4)	53.2 (d)	1.33, m	44.6 (d)
**7**	1.03, m	49.5 (d)	1.15, m	49.7 (d)	1.18, m	50.1 (d)
**8a**	1.51, m	22.6	1.58, m	22.3	1.62, m	23.1
**8b**	1.51, m	(t)	1.11, m	(t)	1.07, m	(t)
**9a**	1.34, m	41.5 (t)	1.77, m	41.0 (t)	1.73, m	41.8 (t)
**9b**	1.34, m		1.38, m		1.37, m	
**10**		73.4 (s)		72.0 (s)		73.0 (s)
**11**	1.30, m	28.7 (d)	1.38, m	29.0 (d)	1.91, m	28.1 (d)
**12 and 13**	0.61, d (6.8) 0.83, d (6.8)	21.7 (q)	0.66, d (6.8)	21.9 (q)	0.89, d (8.4) 0.93, d (7.2)	15.6 (q)
		15.4 (q)	0.89, d (6.8)	15.4 (q)		21.8 (q)
**14**	1.21, s	20.5 (q)	1.26, s	21.0 (q)	1.17, s	20.1 (q)
**15**	2.15, s	29.8 (q)	2.20, s	30.1 (q)	1.18, d (6.4)	23.1 (q)
**C=O**				171.3 (s)		
**Me**			2.04, s	21.3 (q)		

Compounds **3** and **4**, both isolated as optically active white floc with $[\alpha ]_{D}^{20}$ −17 (*c* 0.1, CHCl_3_) and $[\alpha ]_{D}^{20}$ −27 (*c* 0.1, CHCl_3_), respectively, possessed the same molecular formula of C_15_H_28_O_2_ based on the positive HRESIMS data at *m*/*z* 258.2422 and 258.2428, respectively ([M + NH_4_]^+^, calcd. 258.2428). Their IR peaks at 3398 and 3352 cm^−1^ suggested the presence of hydroxy groups. Their ^13^C NMR spectra were similar to those of **1**, with the exception that the carbonyl carbon at *δ*_C_ 212.3 in **1** was replaced by hydroxy-bearing methine carbons at *δ*_C_ 68.6 and 69.7 in **3** and **4**, respectively. The replacements of the C-4 ketone by secondary hydroxy group were supported by the presence of a quartet at *δ*_H_ 3.98 (1H, *J* = 6.4 Hz, assigned to H-4) of **3** and a doublet of quartet at *δ*_H_ 4.00 (1H, *J* = 4.0, 6.4 Hz, assigned to H-4) for **4** in the ^1^H NMR spectrum, which were confirmed from HMBC correlations from H-4 to C-3, C-5, C-6, and C-15. The structures of **3** and **4** were thus deduced as epimers of 4,10-dihydroxyoplopanane. The relative configurations of **3** and **4** were finalized by comparing their spectroscopic features with those of **1** and NOE experiment. The large coupling constants of *J*_1,6_ = 11.6 Hz in **3**/**4** and **3a**/**4a** (acetate of **3**/**4**, Sect. 3 and Tables 2, 3) were used to assign H-1 and H-6 as *α*- and *β*-orientation, respectively. Irradiation of H-4 enhanced signals for H-6 and H-11 in **3a** (acetate of **3**) and **4** allowed H-5 and H-7 as *α*-orientated. Efforts on the determination of the absolute configurations at C-4 in compounds **3** and **4** by using Mosher method failed. Therefore the structures of **3** and **4** were elucidated as depicted and named oplopananol and 4-*epi*-oplopananol, respectively.

**Table 3:** NMR spectroscopic data for compounds **3a**, **4**, and **4a** in CDCl_3_

Position	**3a**		**4**		**4a**	
	*δ*_H_ (*J* in Hz)	*δ*_C_	*δ*_H_ (*J* in Hz)	*δ*_C_	*δ*_H_ (*J* in Hz)	*δ*_C_
**1**	1.38, m	57.6 (d)	1.44, dt (4.0, 12.8)	57.9 (d)	1.43, dt (4.8, 11.6)	57.7 (d)
**2a**	1.70, m	25.4 (t)	1.77, m	26.0 (t)	1.74, m	26.0 (t)
**2b**	1.20, m		1.08, m		1.74, m	
**3a**	1.86, m	24.4 (t)	1.80, m	23.6 (t)	1.80, m	24.2 (t)
**3b**	1.55, m		1.13, m		1.53, m	
**4**	4.98, q (6.4)	73.1 (d)	4.00, dq (4.0, 6.4)	69.7 (d)	5.06, m	72.7 (d)
**5**	1.38, m	46.9 (d)	2.05, m	48.7 (d)	2.12, m	45.2 (d)
**6**	1.14, m	45.1 (d)	0.93, m	46.2 (d)	0.98, m	45.9 (d)
**7**	1.22, m	50.0 (d)	1.21, m	50.1 (d)	1.18, m	49.9 (d)
**8a**	1.59, m	23.3 (t)	1.63, ddt (3.2, 4.0, 13.6)	23.4 (t)	1.63, dq (4.0, 13.6)	23.2 (t)
**8b**	1.03, dt (3.6, 11.6)		1.06, m		1.08, m	
**9a**	1.76, dt (3.6, 12.4)	41.9 (t)	1.76, m	41.9 (t)	1.77, m	41.9 (t)
**9b**	1.35, m		1.37, dt (4.0, 13.2)		1.36, dt (4.0, 12.8)	
**10**		73.0 (s)		73.0 (s)		73.0 (s)
**11**	1.86, m	28.2 (d)	1.93, m (2.4)	28.5 (d)	2.09, m	28.1 (d)
**12 and 13**	0.72, d (7.2) 0.90, d (6.8)	22.0 (q)	0.72, d (6.8) 0.94, d (6.8)	15.7 (q)	0.75, d (6.8) 0.94, d (6.8)	15.7 (q)
		15.5 (q)		21.9 (q)		22.0 (q)
**14**	1.12, s	19.9 (q)	1.14, s	20.1 (q)	1.14, s	20.1 (q)
**15**	1.19, d (6.4)	19.6 (q)	1.12, d (6.4)	17.0 (q)	1.16, d (6.4)	13.8 (q)
**16**		170.9 (s)				170.6 (s)
**17**	2.01, s	21.3 (q)			2.01, s	21.5 (q)

On the basis of NMR, MS, optical rotation data and comparison with literature values, the known sesquiterpenoids were elucidated as oplopanone (**5**) [11], taiwaninone A (**6**) [12], T-muurolol (**7**) [13], *α*-cadinol (**8**) [14], and clovane-2*β*,9*α*-diol (**9**) [15]. Naturally occurring oplopanane sesquiterpenoids are rarely reported [16, 17]. Literature searching showed that there were no more than 20 such type sesquiterpenoids reported up to now, distributed among the families of Alismataceae [16, 17], Araliaceae [18], Araceae [19], Asteraceae [20], Chloranthaceae [21], Cyperaceae [22], Magnoliaceae [23], Meliaceae [24], Schisandraceae [25], Salicaceae [26], and Zingiberaceae [27]. In this study, six oplopananes (**1**–**6**), including four new (**1**–**4**) and one 3,5-*seco*-oplopanane (**6**), were discovered from the rhizomes of *H. occulta*. In addition three new oplopananes (**2a**–**4a**) were also obtained by chemical transformation. These results indicated that *H. occulta* was a rich source of novel natural products.

## Experimental Section

### General

Optical rotations were recorded on a 241 polarimeter (Perkin-Elmer). Infrared (IR) spectra were obtained with a FTS 165-IR instrument (Bio-Rad, USA). NMR spectra were acquired on a Varian INOVA-400 FT-NMR spectrometer (USA). HRESIMS were measured on a Bruker APEX II spectrometer. Sephadex LH-20 (Amersham Biosciences) and silica gel (200**–**300 mesh, Qingdao Haiyang Chemical Co., Ltd) were used for column chromatography (CC), whereas TLC analyses were carried out with glass plates pre-coated with silica gel and the spots were visualized by spraying with 98 % H_2_SO_4_/EtOH in (5/95, v/v) followed by heating. All solvents used were analytical grade.

### Plant Materials

The rhizomes of *H. occulta* Lours (Araceae), collected from Guangxi in China, were purchased from Lanzhou Fuxinghou Herbal Medicines Ltd. Co. in February 2007. The materials were identified by Dr. Huan-Yang Qi at Lanzhou Institute of Chemical Physics (LICP), and a voucher specimen (ZY2007H001) was deposited at the herbarium of LICP.

### Extraction and Isolation

The air-dried rhizomes (13.0 kg) of *H. occulta* were powdered and extracted with 88 % ethanol/water (v/v) at 60 °C (12 h × 3). After dried in vacuum, the residue (410 g) was suspended in water (1.5 L) and applied to a liquid–liquid partitioning against petroleum ether (PE), EtOAc, and *n*-BuOH (each 1.0 L × 3) continuously. The dried PE part (257 g) was chromatographed over silica gel (1.5 kg), using gradient PE/acetone (v/v, from 80:1 to 1:1, each about 8.0 L) to yield eleven fractions (A1–A11). Fraction A2 (55 g) was subjected to silica gel CC eluting with CHCl_3_/PE gradient system to afford compound **8** (18.2 mg). Fraction A3 (21 g) was purified over silica gel with PE/CHCl_3_ (v/v, 1:1) to afford **7** (4.5 mg). Fraction A5 (34 g) was fractionated consecutively over silica gel and Sephadex LH-20 (CHCl_3_/MeOH, 1:1, v/v) to yield compound **5** (4.5 mg). Fraction A6 (12 g) was chromatographed on a silica gel column eluting with PE/acetone (v/v, 10:1, 8:1, 5:1, 3:1, and 1:1) to afford compound **1** (5.1 mg). Fraction A7 (15 g) was fractionated consecutively over silica gel with PE/acetone (8:1) and Sephadex LH-20 (CHCl_3_/MeOH, 1:1, v/v) to yield **2** (7.8 mg), **3** (23.1 mg), **4** (17.7 mg), **6** (17.8 mg), and **9** (2.1 mg).

#### 7-*Epi*-oplopanone (**1**)

Colorless oil; $[\alpha ]_{D}^{20}$–54 (*c* 0.07, CHCl_3_); IR (neat) *v*_max_ 3382, 2926, 2857, 1705, 1458, 1383, 1119, 1094, 1027 cm^−1^; ^1^H NMR (CDCl_3_, 400 MHz) and ^13^C NMR (CDCl_3_, 100 MHz) (Table 1); HRESIMS *m*/*z* [M + NH_4_]^+^ 256.2272 (calcd for C_15_H_30_O_2_N, 256.2271).

#### 5,7-*Diepi*-2*α*-hydroxyoplopanone (**2**)

Colorless oil; $[\alpha ]_{D}^{20}$ +20 (*c* 0.1, CHCl_3_); IR (neat) *v*_max_ 3392, 2955, 2935, 2871, 1704, 1460, 1380, 1365, 1173, 1121, 1049 cm^−1^; ^1^H NMR (CDCl_3_, 400 MHz) and ^13^C NMR (CDCl_3_, 100 MHz) (Table 2); HRESIMS *m*/*z* [M + Na]^+^ 277.1779 (calcd for C_15_H_26_O_3_Na, 277.1774).

#### Oplopananol (**3**)

White floc; $[\alpha ]_{D}^{20}$–17 (*c* 0.1, CHCl_3_); IR (neat) *v*_max_ 3398, 2959, 2933, 1461, 1370, 1129 cm^−1^; ^1^H NMR (CDCl_3_, 400 MHz) and ^13^C NMR (CDCl_3_, 100 MHz) (Table 2); HRESIMS *m*/*z* [M + NH_4_]^+^ 258.2422 (calcd for C_15_H_32_O_2_N, 258.2428).

#### 4-*Epi*-oplopananol (**4**)

White floc; $[\alpha ]_{D}^{20}$ –27 (*c* 0.1, CHCl_3_); IR (neat) *v*_max_ 3352, 2956, 2924, 2856, 1571, 1458, 1421, 1365, 1128, 1062, 944 cm^−1^; ^1^H NMR (CDCl_3_, 400 MHz) and ^13^C NMR (CDCl_3_, 100 MHz) (Table 3); HRESIMS *m*/*z* [M + NH_4_]^+^ 258.2428 (calcd for C_15_H_32_O_2_N, 258.2428).

### Acetylation of **2** to **4**

A solution of compounds in a mixture of acetic anhydride–pyridine (1:1) were stirred fully and settled at 25 °C for 12 h. After concentration and storage in *vacuo* compounds **2a**, **3a**, and **4a** were obtained and identified.

#### 5,7-*Diepi*-2*α*-acetoxyoplopanone (**2a**)

Colorless oil; $[\alpha ]_{D}^{20}$ +7 (*c* 0.2, CHCl_3_); IR (neat) *v*_max_ 3449, 2934, 2871, 1734, 1712, 1370, 1247, 1044, 968 cm^−1^; ^1^H NMR (CDCl_3_, 400 MHz) and ^13^C NMR (CDCl_3_, 100 MHz) (Table 2); HRESIMS *m*/*z* [M + Na]^+^ 319.1883 (calcd for C_17_H_28_O_4_Na, 319.1880).

#### 4-Acetoxyoplopananol (**3a**)

Colorless oil; $[\alpha ]_{D}^{20}$–41 (*c* 0.2, CHCl_3_); UV (CHCl_3_) *λ*_max_(log ε) 246 (1.56) nm; IR (neat) *v*_max_ 3423, 2960, 2935, 2892, 1736, 1458, 1375, 1247, 1134, 1033, 930 cm^−1^; ^1^H NMR (CDCl_3_, 400 MHz) and ^13^C NMR (CDCl_3_, 100 MHz) (Table 3); HRESIMS *m*/*z* [M + Na]^+^ 305.2091 (calcd for C_17_H_30_O_3_Na, 305.2087).

#### 4-*Epi*-acetoxyoplopananol (**4a**)

White floc; $[\alpha ]_{D}^{20}$ –10 (*c* 0.2, CHCl_3_); UV (CHCl_3_) *λ*_max_(log ε) 246 (1.48) nm; IR (neat) *v*_max_ 3420, 2858, 2935, 2872, 1732, 1374, 1248, 1126, 1035, 953 cm^−1^; ^1^H NMR (CDCl_3_, 400 MHz) and ^13^C NMR (CDCl_3_, 100 MHz) (Table 3); HRESIMS *m*/*z* [M + Na]^+^ 305.2093 (calcd for C_17_H_30_O_3_Na, 305.2087).

## Electronic supplementary material

Below is the link to the electronic supplementary material.

Supplementary material 1 (DOC 1836 kb)
