# Supplementary material for: Sesquiterpenoids from the Rhizomes of Homalomenaocculta
Source: Nat Prod Bioprospect. 2016 Jul 5;6(4):211–6. doi: 10.1007/s13659-016-0104-8 (PMC4940255; doi:10.1007/s13659-016-0104-8)
Supplement: Supplementary file 1 — Supplementary material 1 (DOC 1836 kb) [file 13659_2016_104_MOESM1_ESM.doc]

**Sesquiterpenoids from the Rhizomes of *Homalomena* *occulta***

Jun-Li Yang ^a^, Ya-Min Zhao ^b^ and Yan-Ping Shi ^a, b^ ,[[1]](#footnote-2)*

^a^ *Key Laboratory of Chemistry of Northwestern Plant Resources and Key Laboratory for Natural Medicine of Gansu Province, Lanzhou Institute of Chemical Physics, Chinese Academy of Sciences, Lanzhou 730000, People’s Republic of China.*

^b^ *State Key Laboratory of Applied Organic Chemistry, Lanzhou University, Lanzhou 730000, People’s Republic of China*

**For Compound 1**


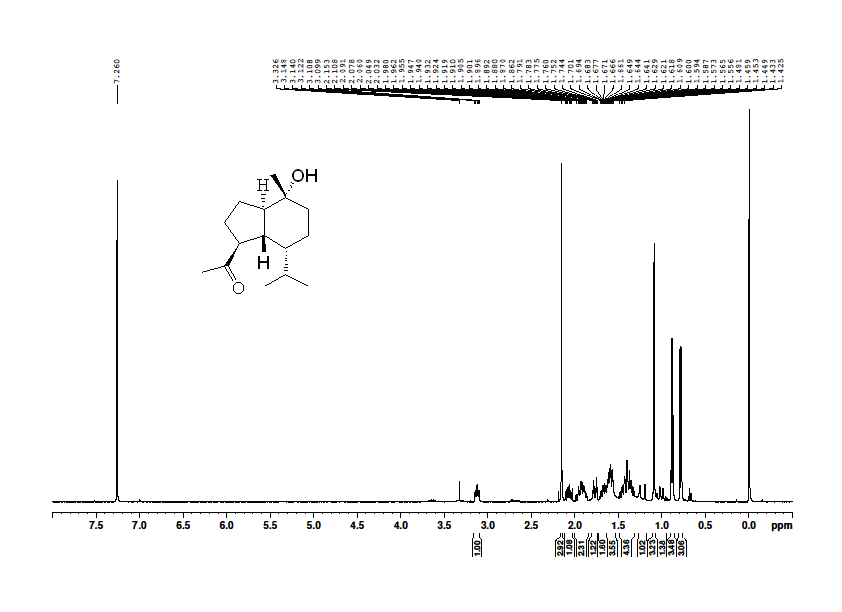


**Figure S1.** ^1^H NMR Spectrum of **1** (400MHz, CDCl_3_)


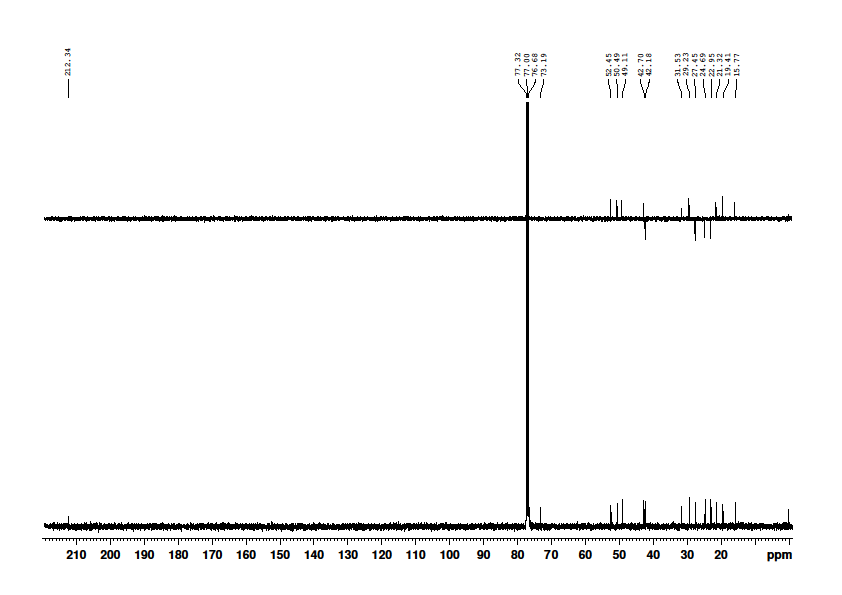


**Figure S2.** ^13^C NMR and DEPT-135 Spectra of **1** (100MHz, CDCl_3_)


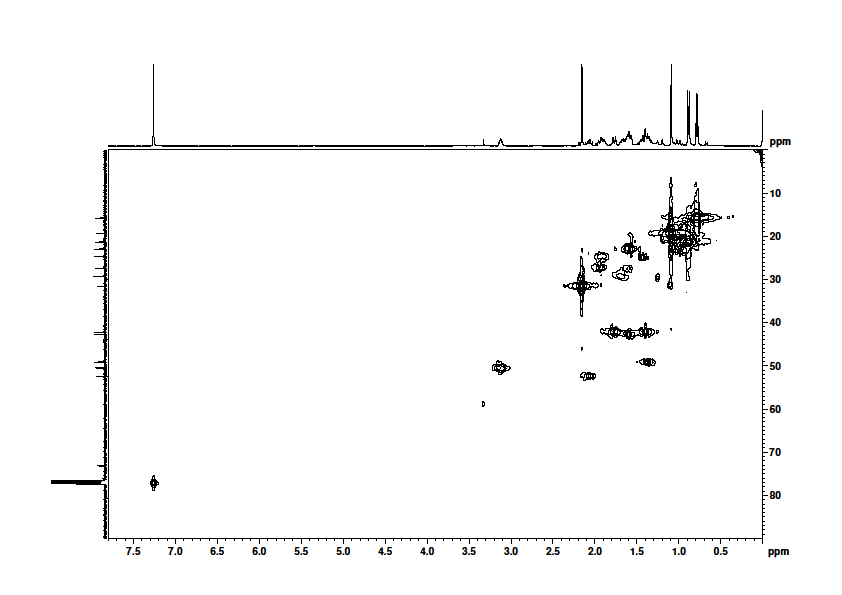


**Figure S3.** HSQC Spectrum of **1** (400MHz, CDCl_3_)


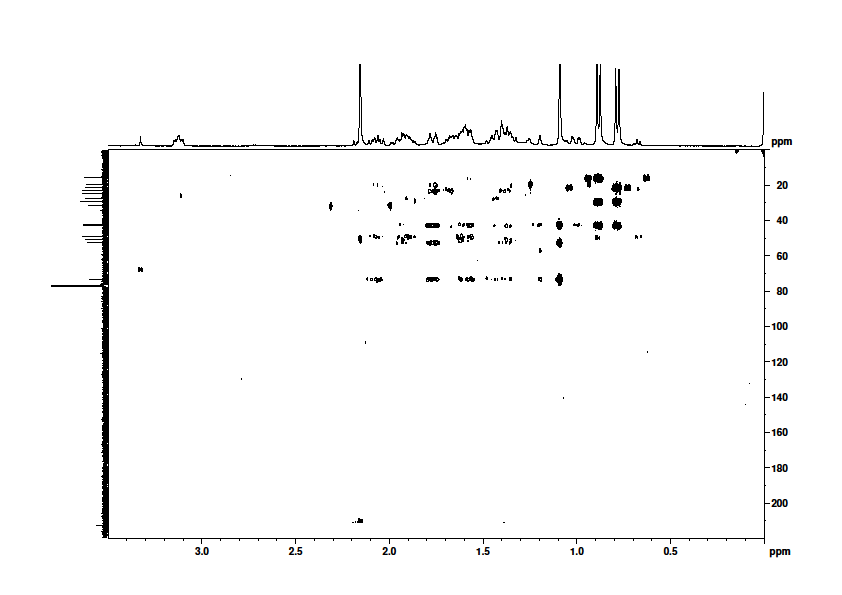


**Figure S4.** HMBC Spectrum of **1** (400MHz, CDCl_3_)


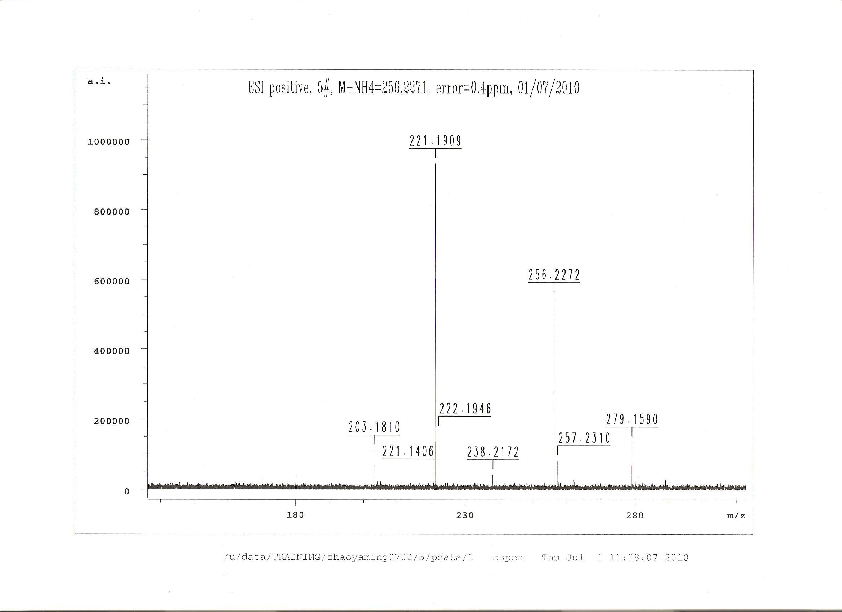


**Figure S5.** HRESIMS Spectrum of **1**

**For Compound 2**


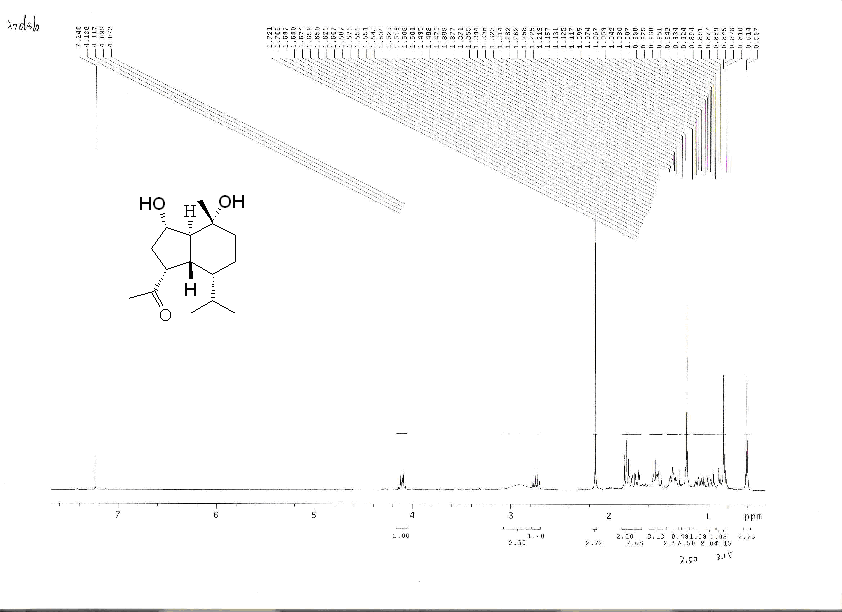


**Figure S6.** ^1^H NMR Spectrum of **2** (400MHz, CDCl_3_)


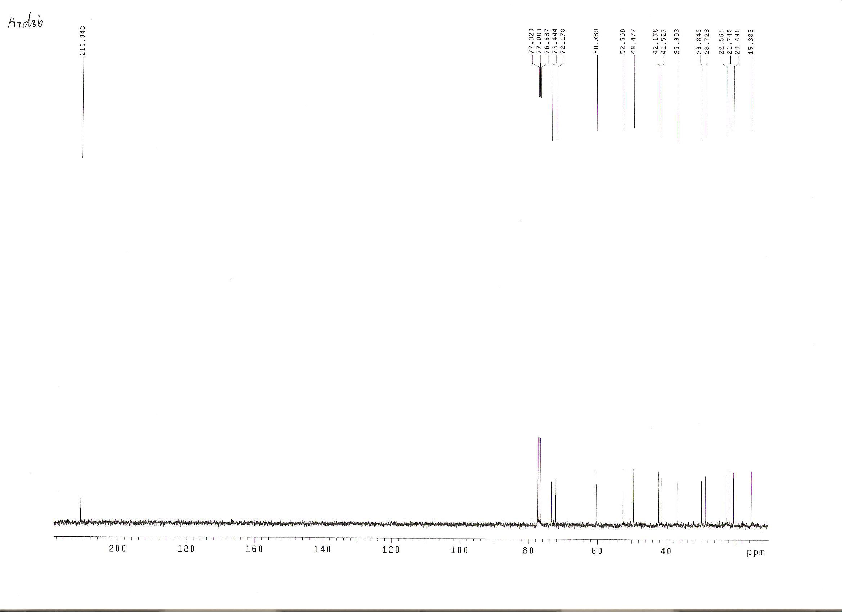


**Figure S7.** ^13^C NMR Spectrum of **2** (100MHz, CDCl_3_)


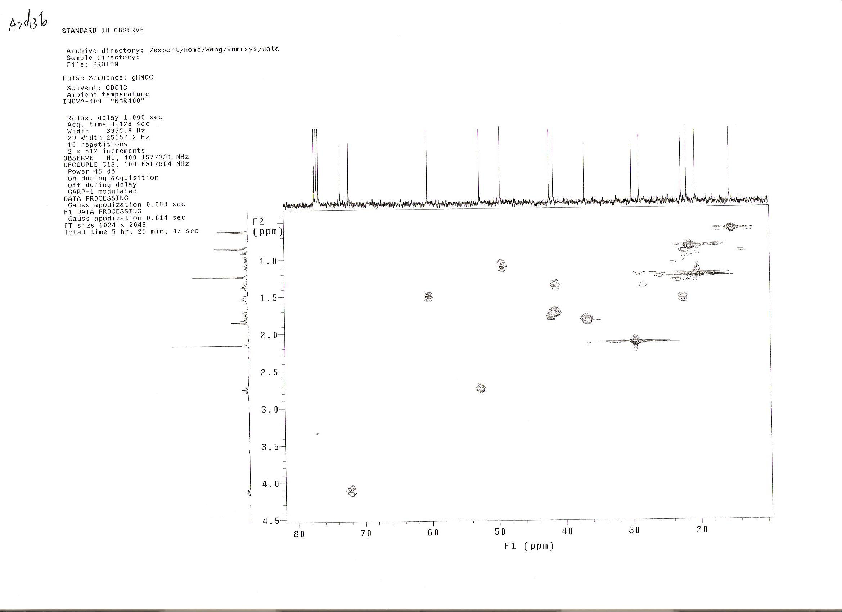


**Figure S8.** HSQC Spectrum of **2** (100MHz, CDCl_3_)


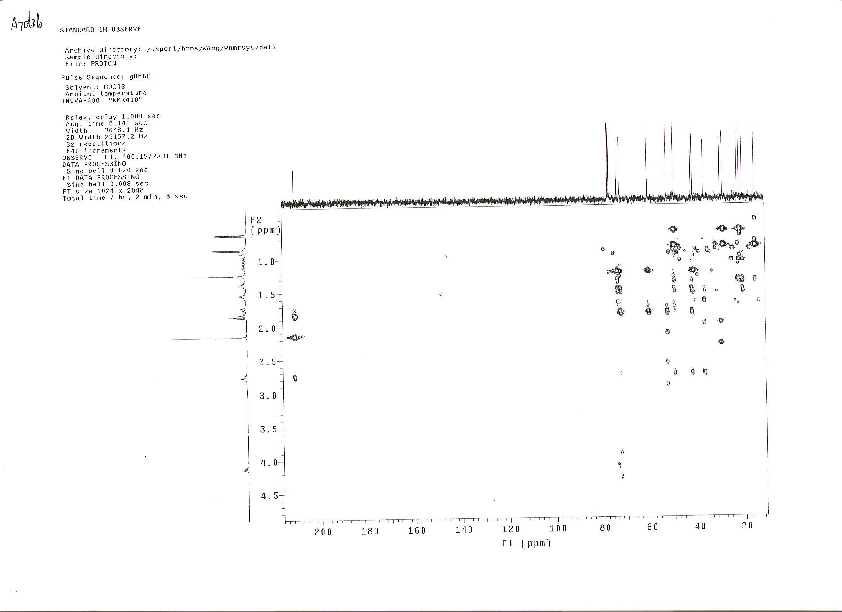


**Figure S9.** HMBC Spectrum of **2** (100MHz, CDCl_3_)


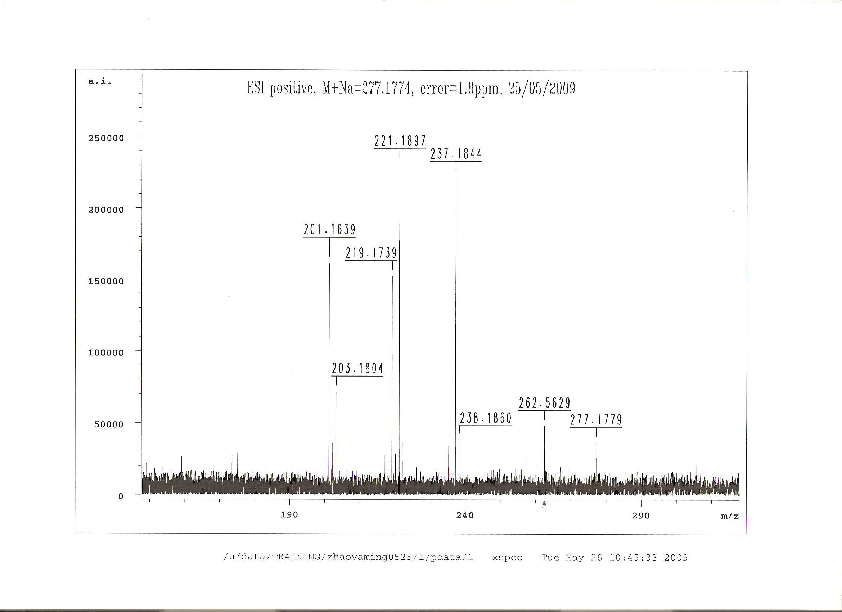


**Figure S10.** HRESIMS Spectrum of **2**

**For Compound 2a**


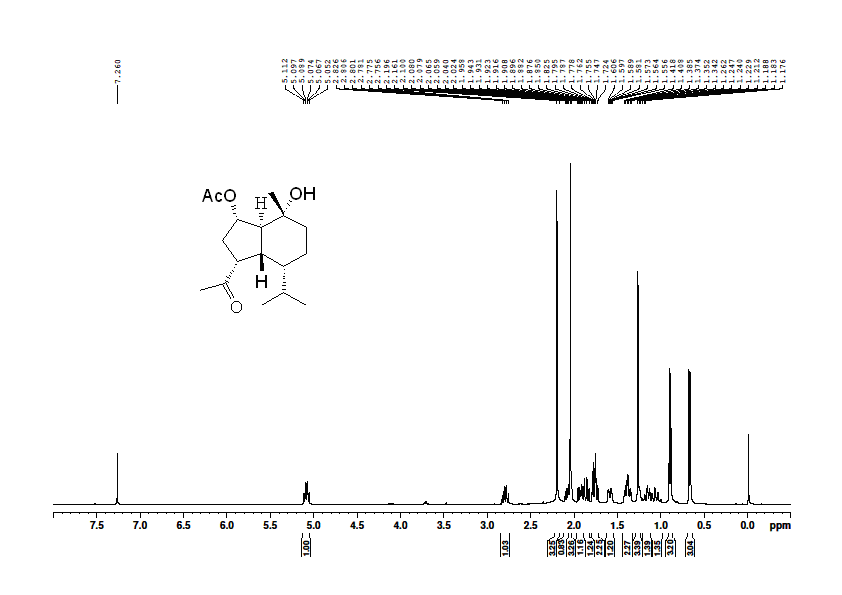


**Figure S11.** ^1^H NMR Spectrum of **2a** (400MHz, CDCl_3_)


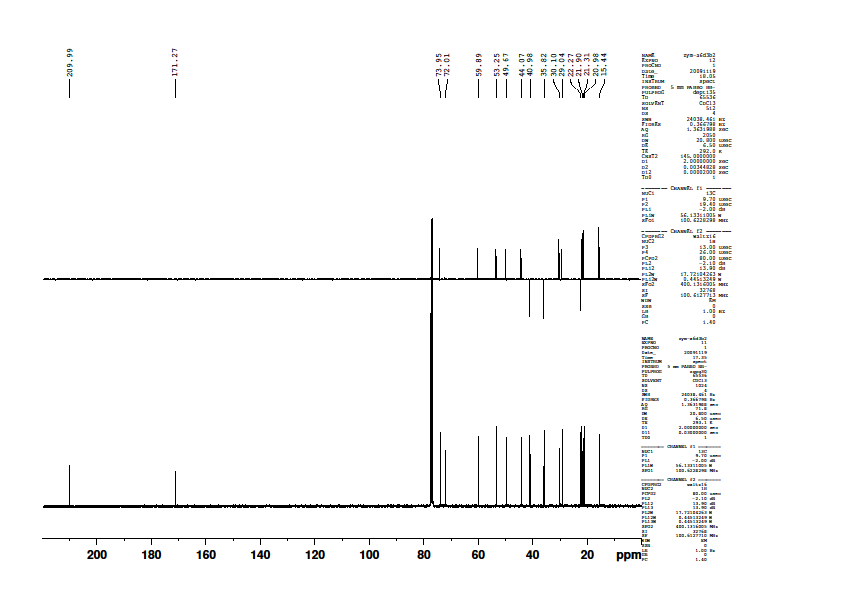


**Figure S12.** ^13^C NMR and DEPT-135 Spectra of **2a** (100MHz, CDCl_3_)


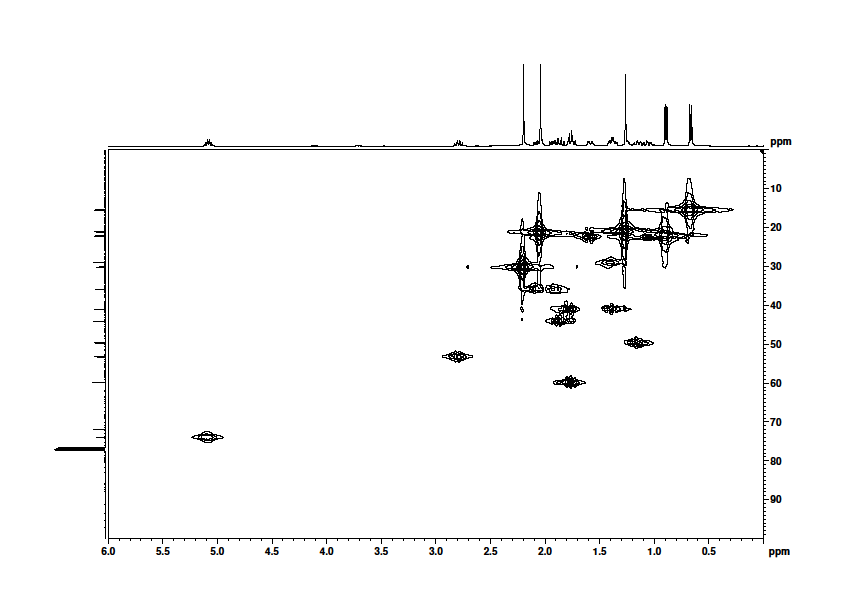


**Figure S13.** HSQC Spectrum of **2a** (400MHz, CDCl_3_)


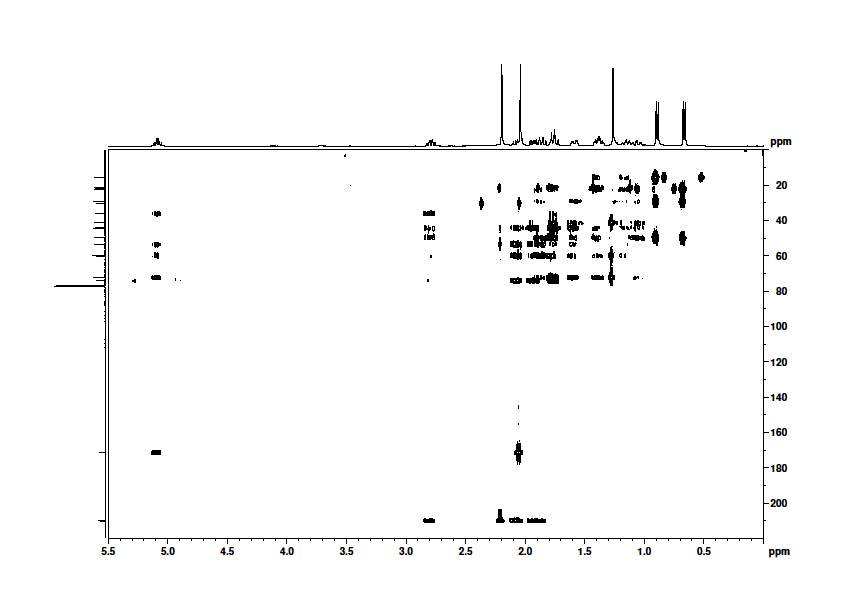


**Figure S14.** HMBC Spectrum of **2a** (400MHz, CDCl_3_)


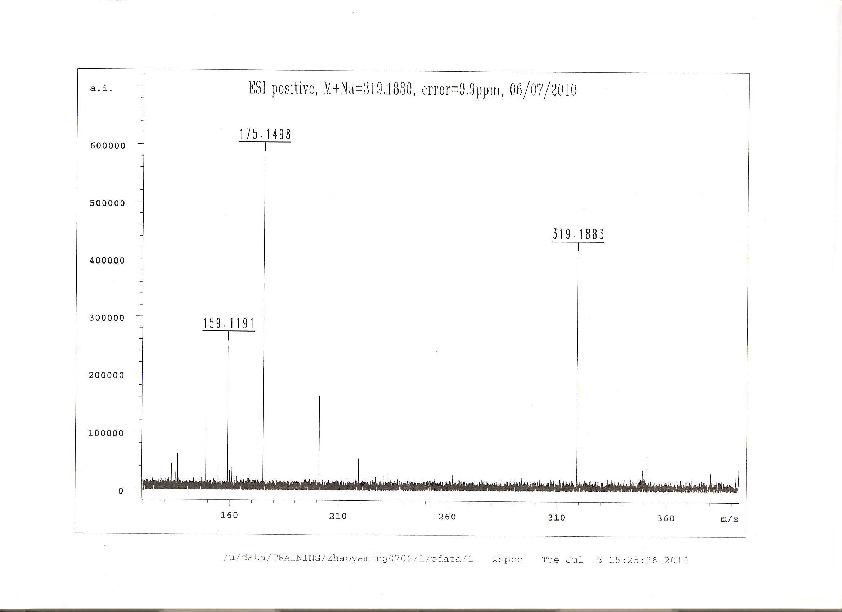


**Figure S15.** HRESIMS Spectrum of **2a**


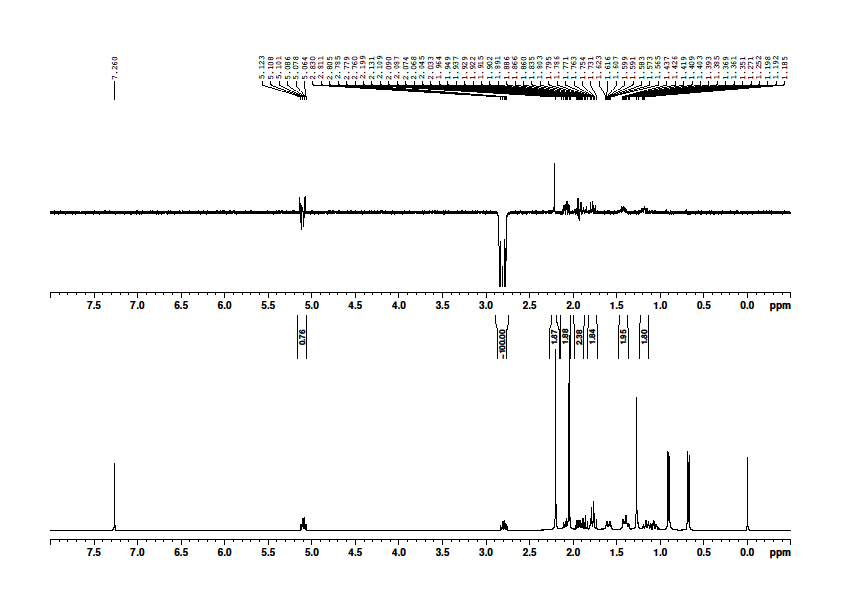


**Figure S16.** NOE-1 Spectrum of **2a** (400MHz, CDCl_3_) (H-5: H-2, H-7)


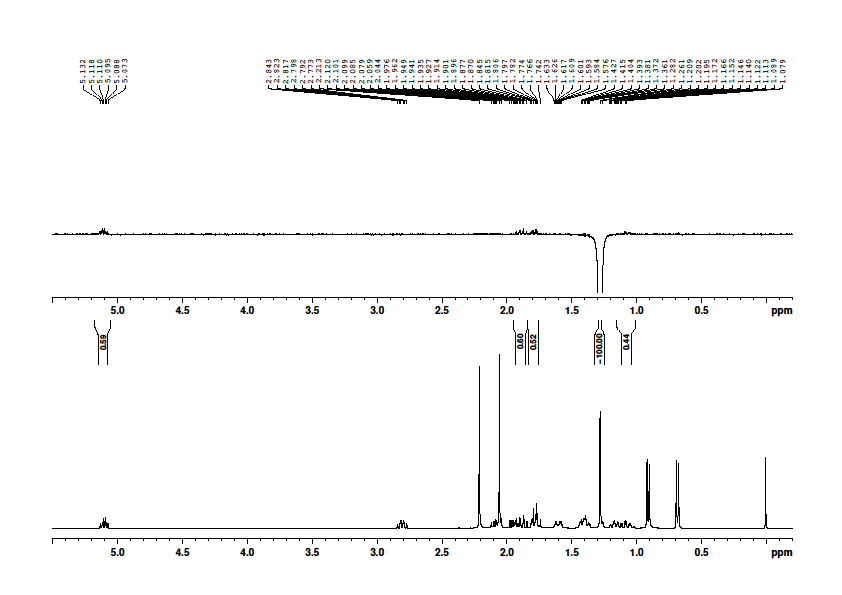


**Figure S17.** NOE-2 Spectrum of **2a** (400MHz, CDCl_3_)

(H3-14: H-2, H-6; )

**For Compound 3**


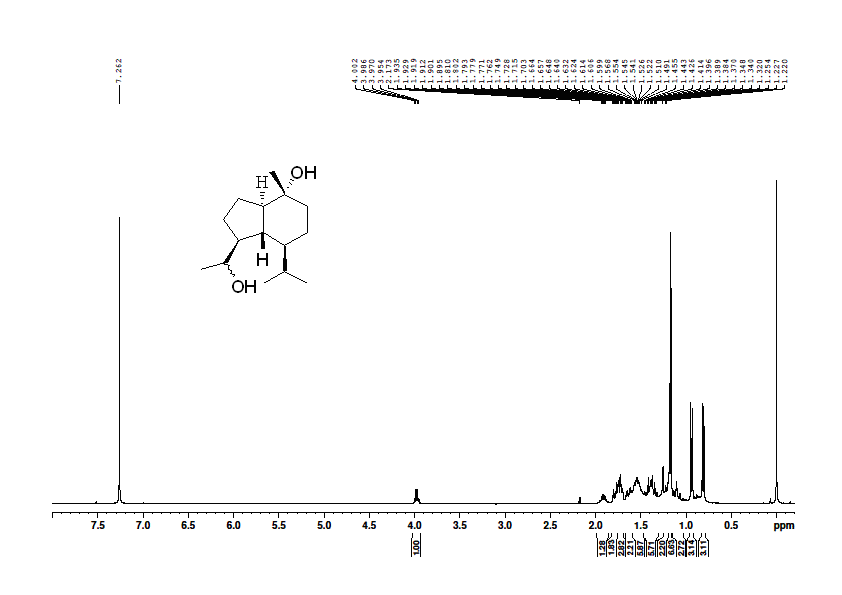


**Figure S18.** ^1^H NMR Spectrum of **3** (400MHz, CDCl_3_)


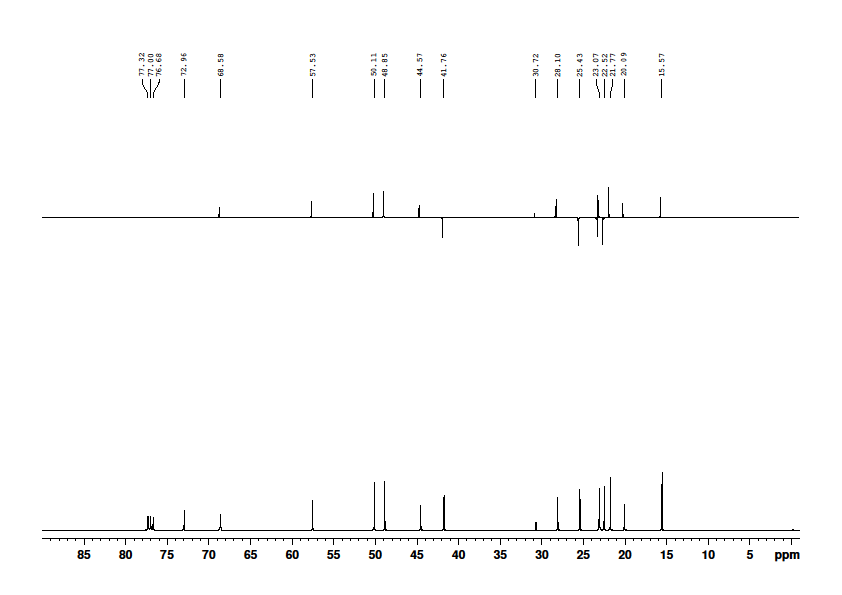


**Figure S19.** ^13^C NMR and DEPT-135 Spectra of **3** (100MHz, CDCl_3_)


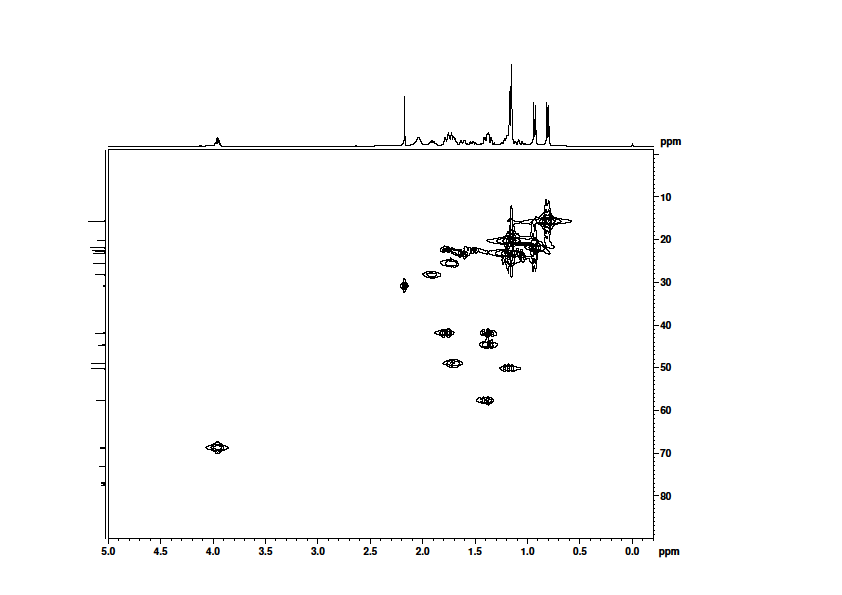


**Figure S20.** HSQC Spectrum of **3** (400MHz, CDCl_3_)


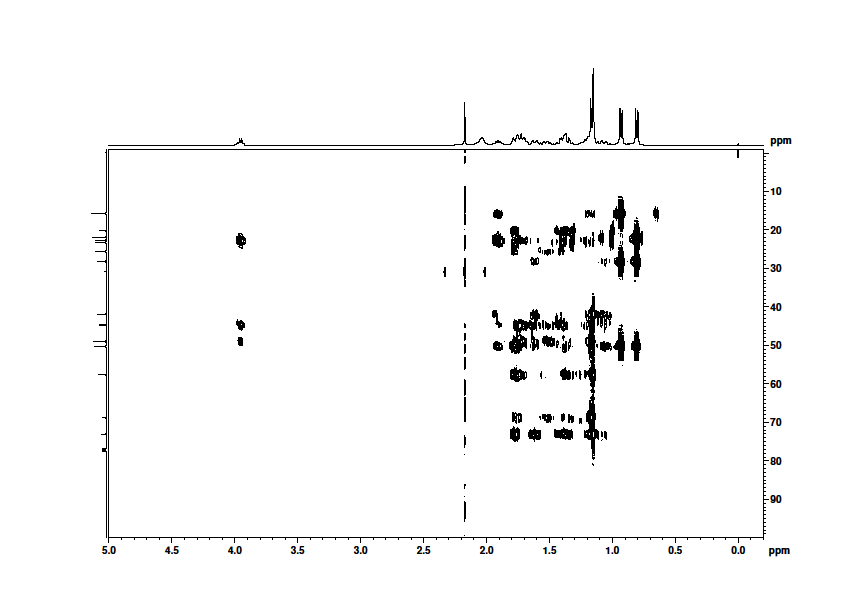


**Figure S21.** HMBC Spectrum of **3** (400MHz, CDCl_3_)


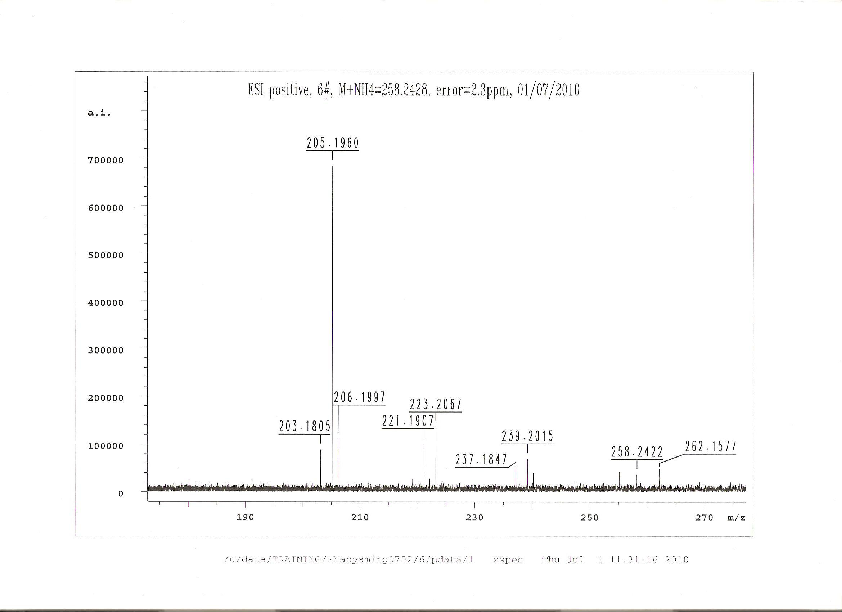


**Figure S22.** HRESIMS Spectrum of **3**


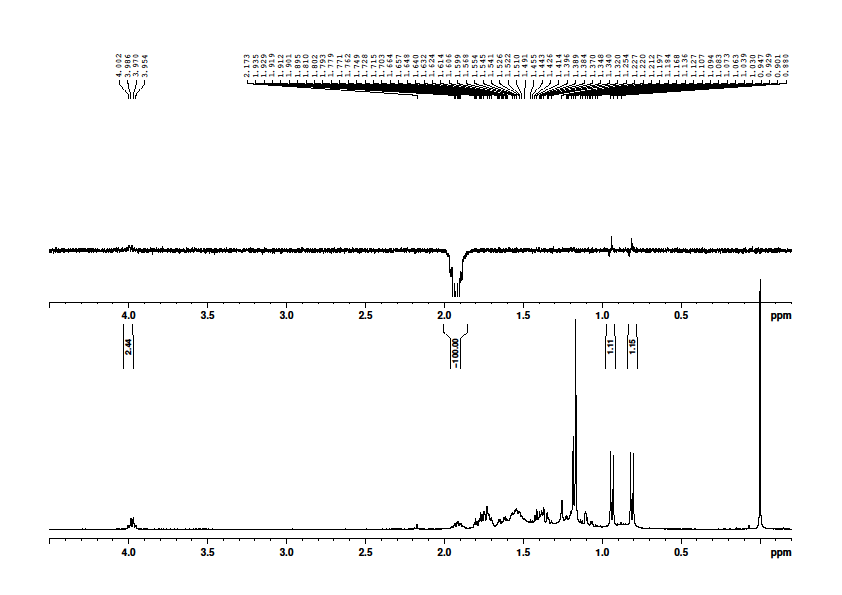


**Figure S23.** NOE Spectrum of **3** (400MHz, CDCl_3_)

**For Compound 3a**


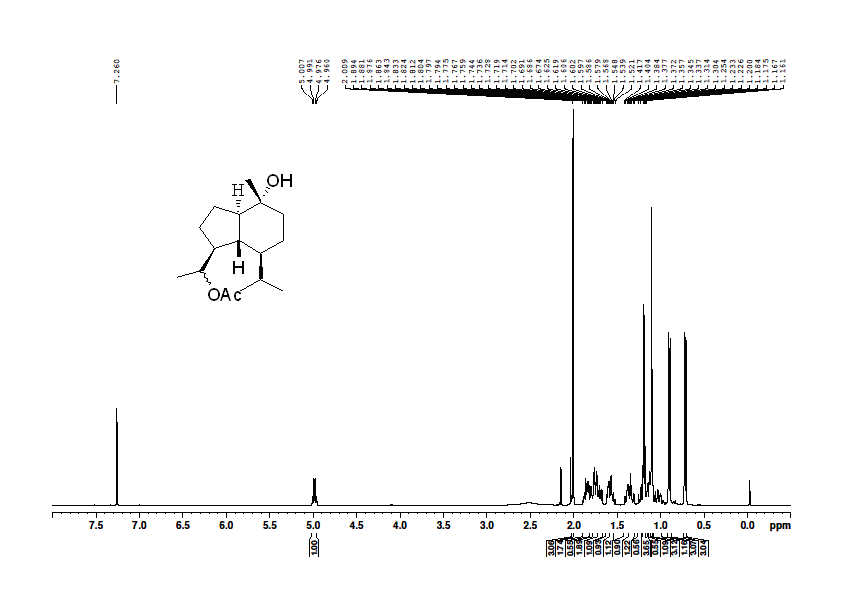


**Figure S24.** ^1^H NMR Spectrum of **3a** (400MHz, CDCl_3_)


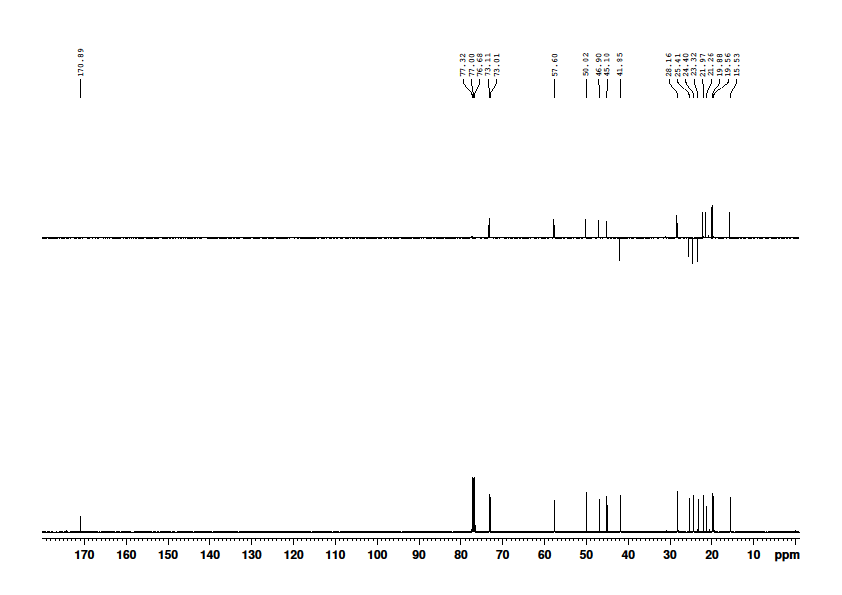


**Figure S25.** ^13^C NMR and DEPT-135 Spectra of **3a** (100MHz, CDCl_3_)


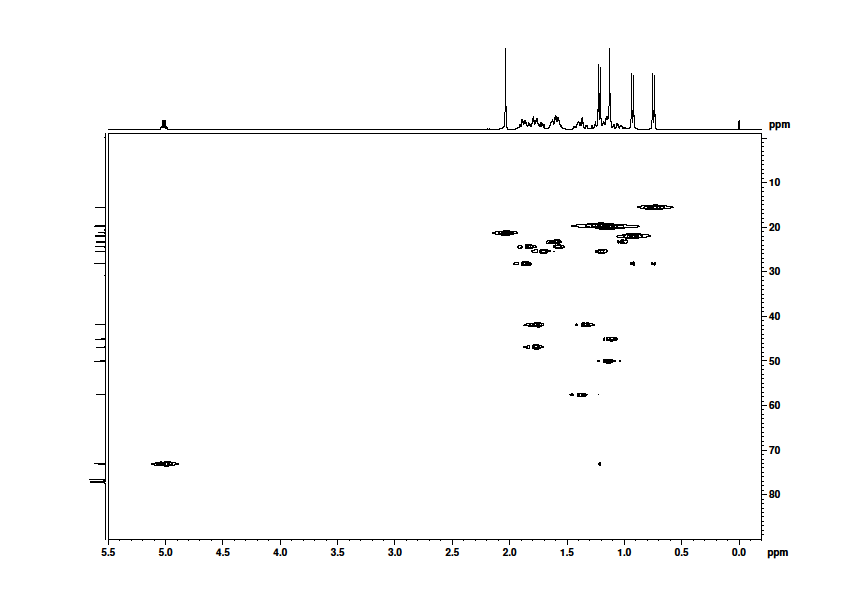


**Figure S26.** HSQC Spectrum of **3a** (400MHz, CDCl_3_)


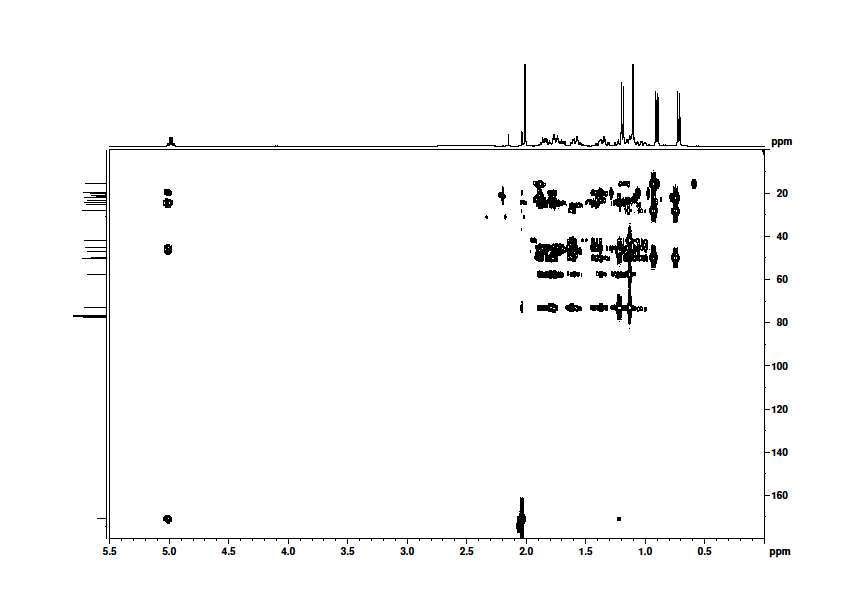


**Figure S27.** HMBC Spectrum of **3a** (400MHz, CDCl_3_)


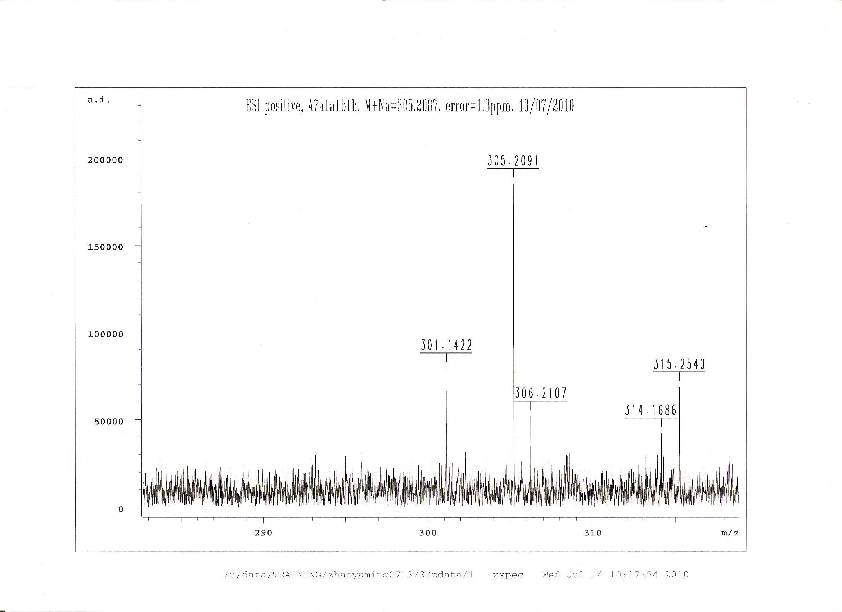


**Figure S28.** HRESIMS Spectrum of **3a**


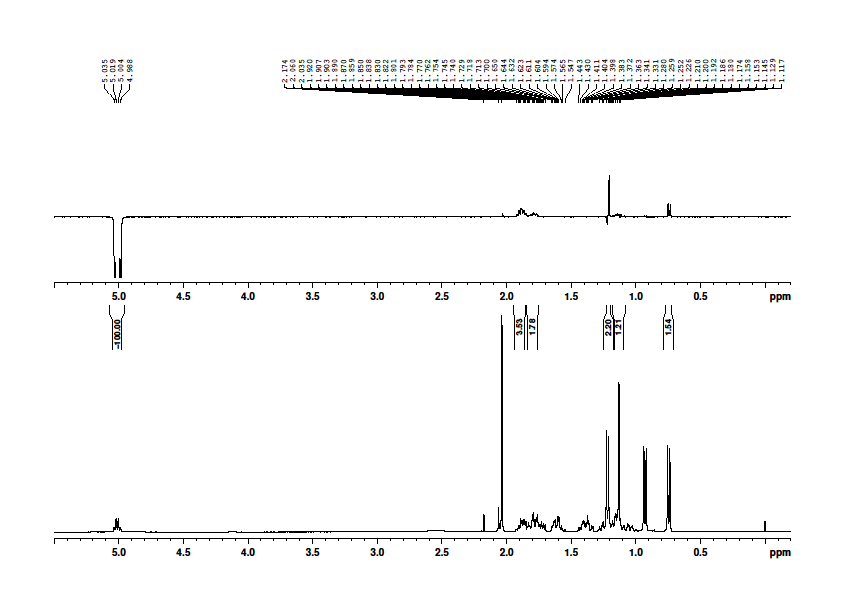


**Figure S29.** NOE Spectrum of **3a** (400MHz, CDCl_3_)

**For Compound 4**


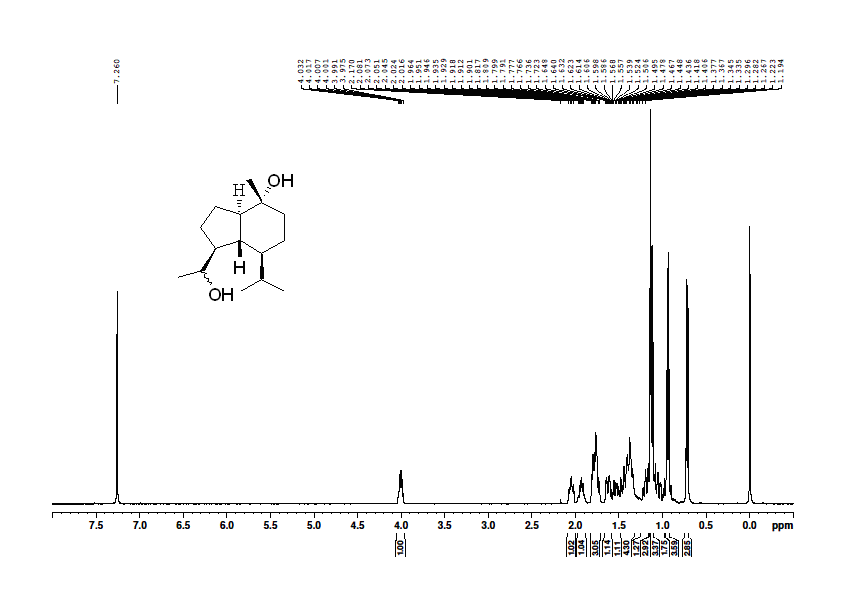


**Figure S30.** ^1^H NMR Spectrum of **4** (400MHz, CDCl_3_)


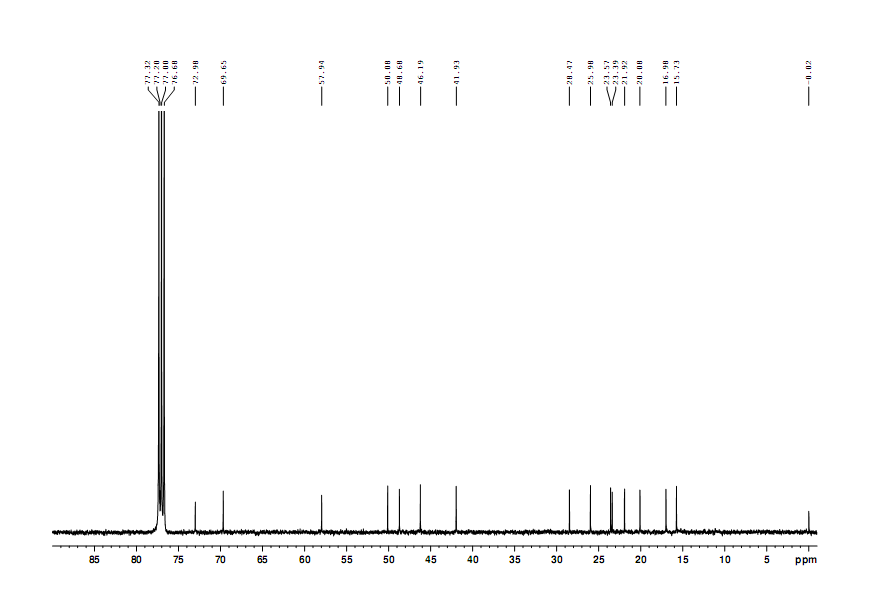


**Figure S31.** ^13^C NMR Spectrum of **4** (100MHz, CDCl_3_)


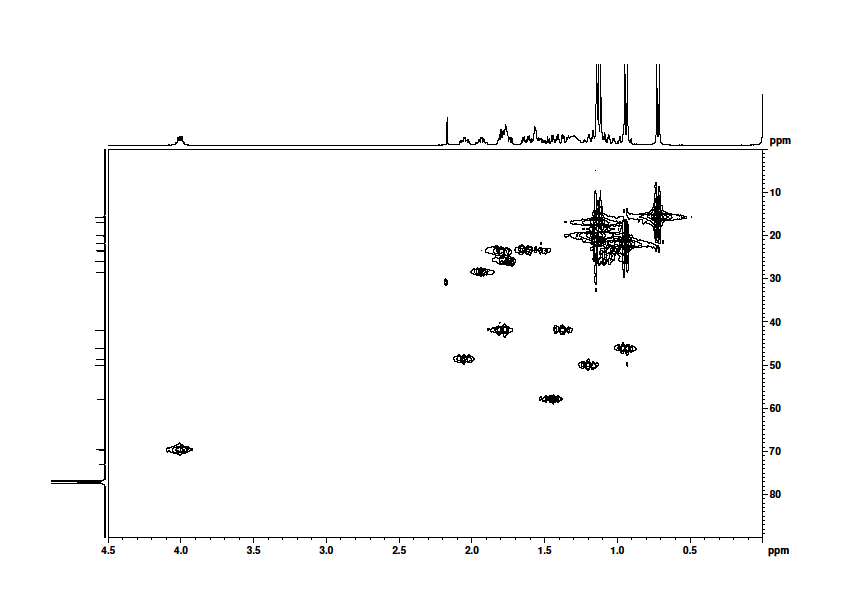


**Figure S32.** HSQC Spectrum of **4** (400MHz, CDCl_3_)


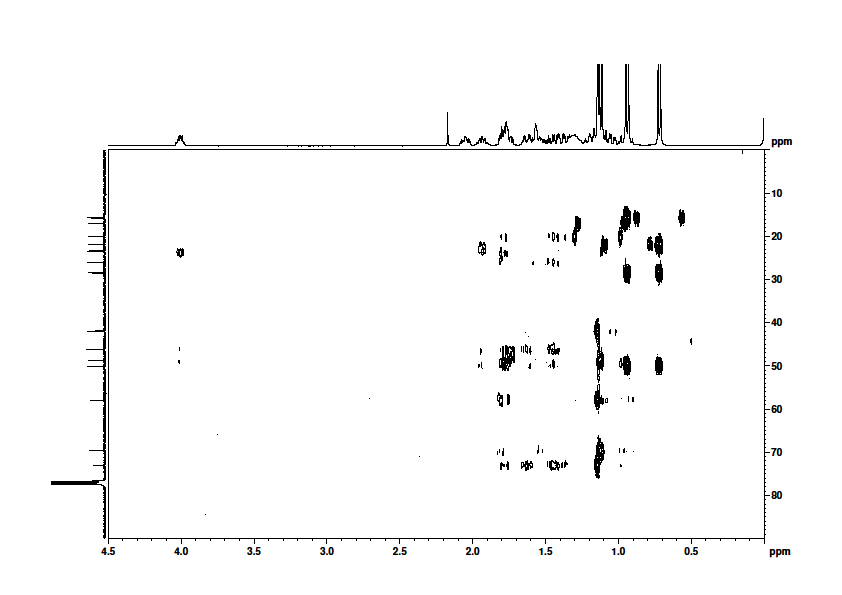


**Figure S33.** HMBC Spectrum of **4** (400MHz, CDCl_3_)


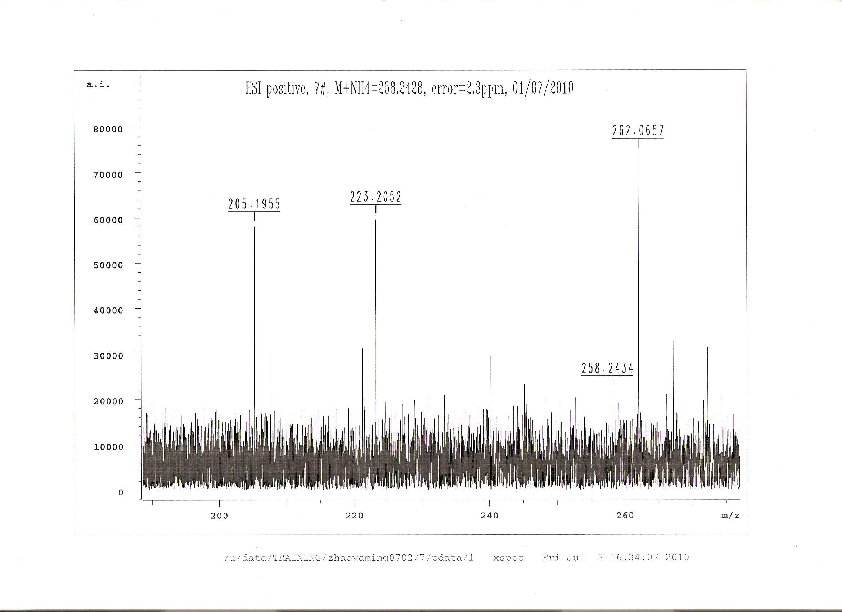


**Figure S34.** HRESIMS Spectrum of **4**


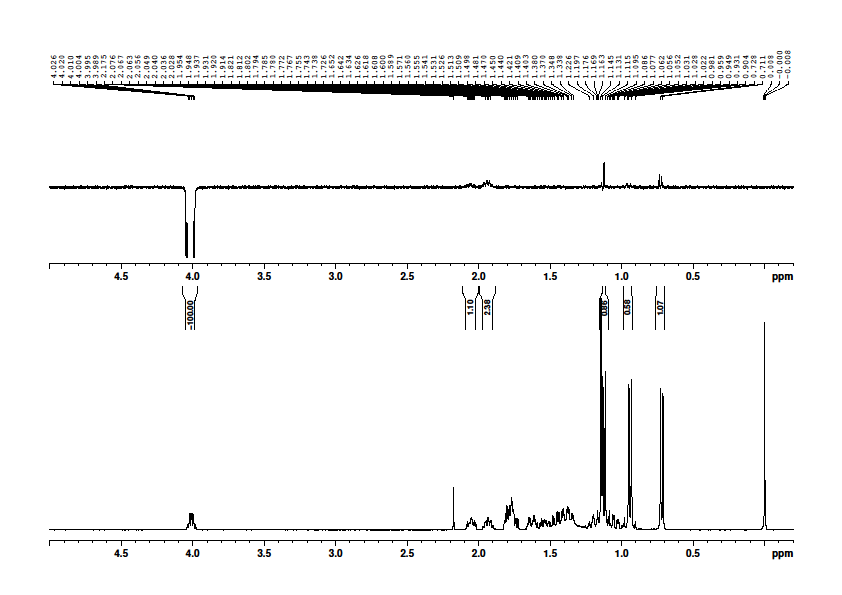


**Figure S35.** NOE Spectrum of **4** (400MHz, CDCl_3_)

**For Compound 4a**


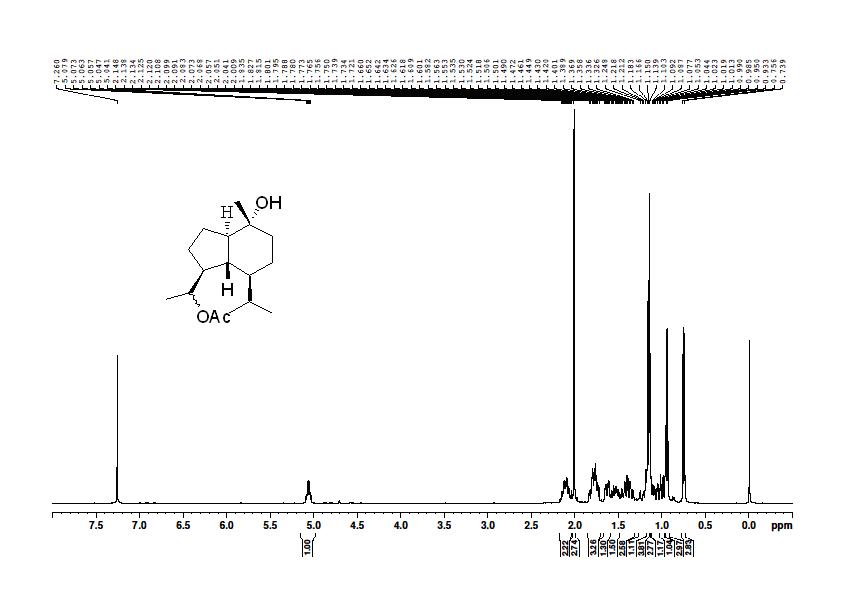


**Figure S36.** ^1^H NMR Spectrum of **4a** (400MHz, CDCl_3_)


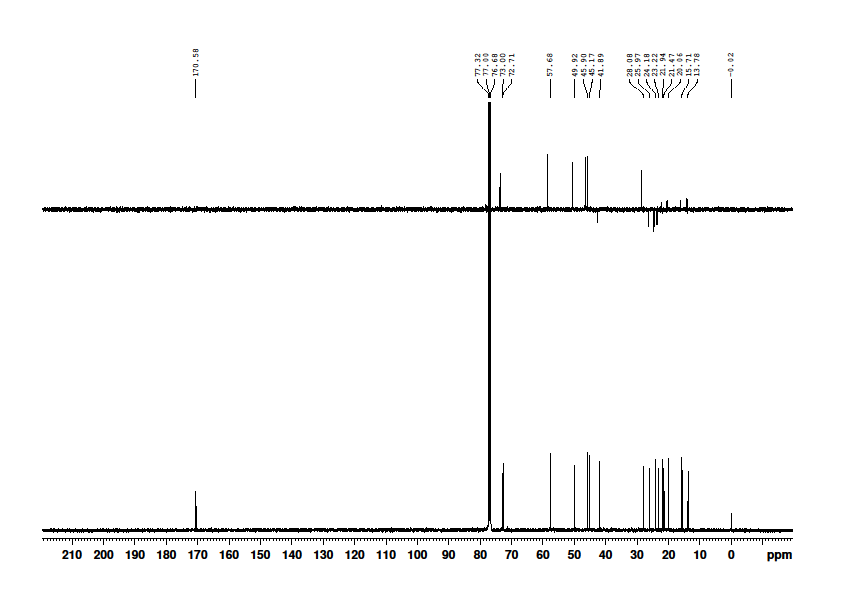


**Figure S37.** ^13^C NMR and DEPT-135 Spectra of **4a** (100MHz, CDCl_3_)


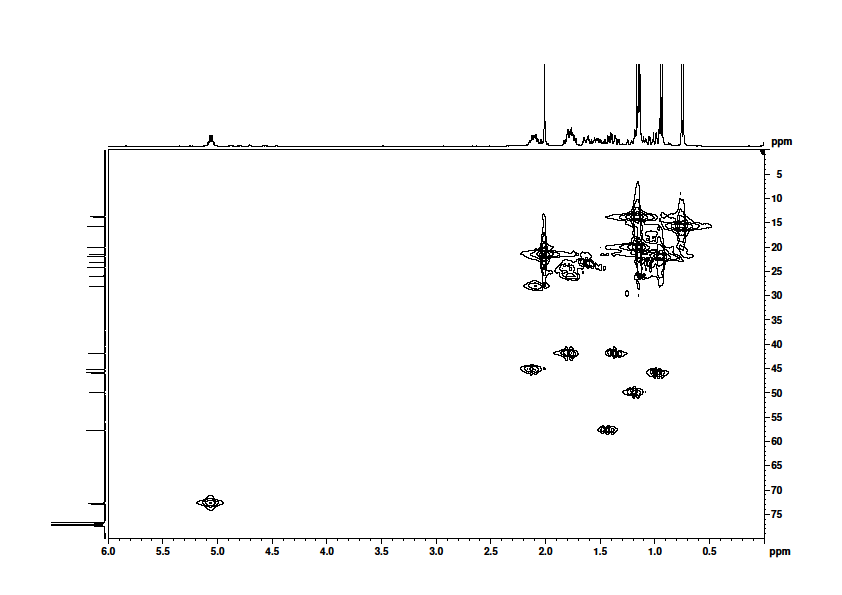


**Figure S38.** HSQC Spectrum of **4a** (400MHz, CDCl_3_)


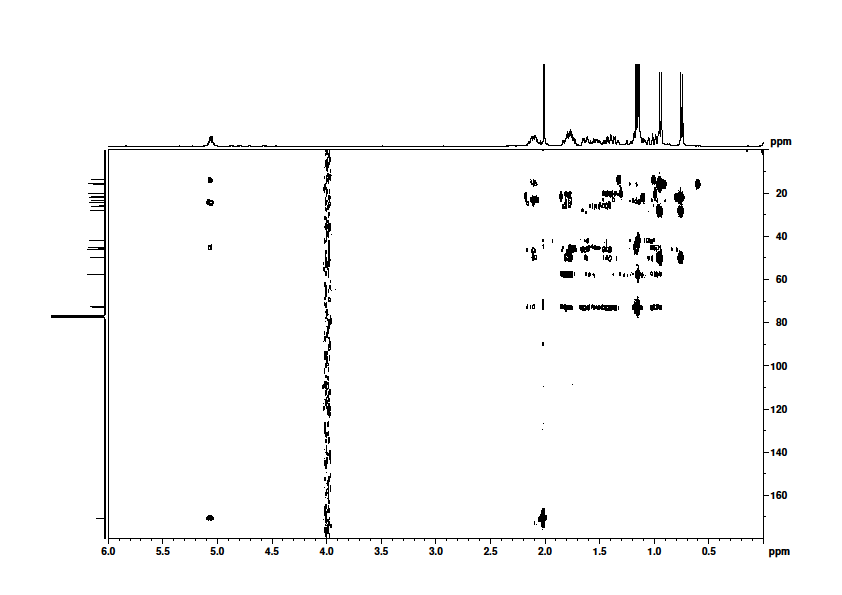


**Figure S39.** HMBC Spectrum of **4a** (400MHz, CDCl_3_)


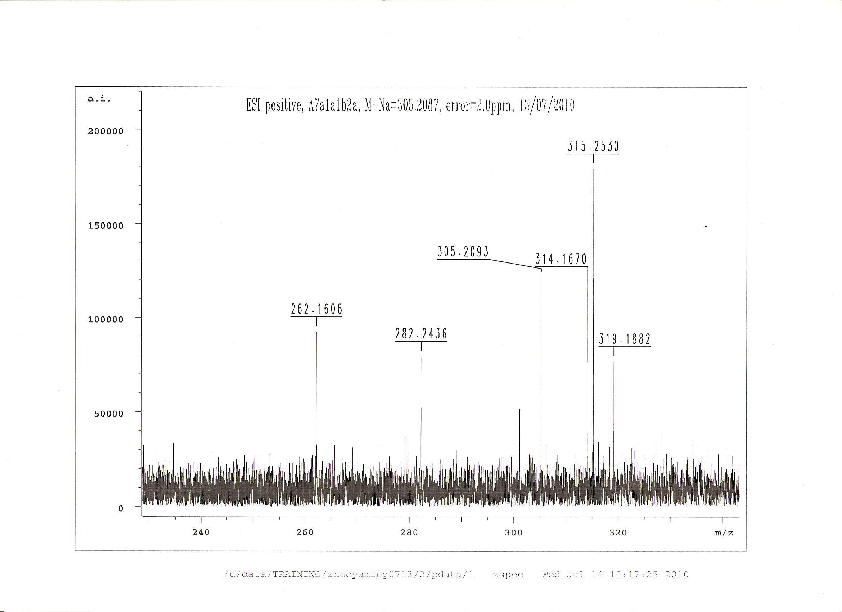


**Figure S40.** HRESIMS Spectrum of **4a**

1. ^*^ Corresponding author, Tel.: +86-931-4968208; Fax: +86-931-4968094. *E-mail address*: [shiyp@licp.cas.cn](mailto:shiyp@licp.cas.cn) (Y.-P. Shi) [↑](#footnote-ref-2)
